# Declines in Pneumonia and Meningitis Hospitalizations in Children Under 5 Years of Age After Introduction of 10-Valent Pneumococcal Conjugate Vaccine in Zambia, 2010–2016

**DOI:** 10.1093/cid/ciz456

**Published:** 2019-09-05

**Authors:** Evans M Mpabalwani, Chileshe Lukwesa-Musyani, Akakambama Imamba, Ruth Nakazwe, Belem Matapo, Chilweza M Muzongwe, Trust Mufune, Elizabeth Soda, Jason M Mwenda, Chelsea S Lutz, Tracy Pondo, Fernanda C Lessa

**Affiliations:** 1 University of Zambia, School of Medicine, Department of Pediatrics & Child Health Unit, Ministry of Health, Ndeke House, Lusaka, Zambia; 2 Lusaka Children’s Hospital Unit, Ministry of Health, Ndeke House, Lusaka, Zambia; and; 3 Microbiology Laboratory Unit, Ministry of Health, Ndeke House, Lusaka, Zambia; University Teaching Hospitals; 4 World Health Organization Zambia Unit, Ministry of Health, Ndeke House, Lusaka, Zambia; 5 Department of Monitoring and Evaluation, Public Health & Research, Health Management Information System Unit, Ministry of Health, Ndeke House, Lusaka, Zambia; 6 Division of Bacterial Diseases, Centers for Disease Control and Prevention, Atlanta, Georgia; 7 World Health Organization Regional Office for Africa, Brazzaville, Republic of Congo; 8 Immunization Services Division, Centers for Disease Control and Prevention, Atlanta, Georgia; 9 Oak Ridge Institute for Science and Education, United States Department of Energy, Washington, DC

**Keywords:** pneumococcus, pneumonia, meningitis, pneumococcal conjugate vaccine, Zambia

## Abstract

**Background:**

Pneumococcus is a leading cause of pneumonia and meningitis. Zambia introduced a 10-valent pneumococcal conjugate vaccine (PCV10) in July 2013 using a 3-dose primary series at ages 6, 10, and 14 weeks with no booster. We evaluated the impact of PCV10 on meningitis and pneumonia hospitalizations.

**Methods:**

Using hospitalization data from first-level care hospitals, available at the Ministry of Health, and from the largest pediatric referral hospital in Lusaka, we identified children aged <5 years who were hospitalized with pneumonia or meningitis from January 2010–December 2016. We used time-series analyses to measure the effect of PCV10 on monthly case counts by outcome and age group (<1 year, 1–4 years), accounting for seasonality. We defined the pre- and post-PCV10 periods as January 2010–June 2013 and July 2014–December 2016, respectively.

**Results:**

At first-level care hospitals, pneumonia and meningitis hospitalizations among children aged <5 years accounted for 108 884 and 1742 admissions in the 42 months pre-PCV10, respectively, and 44 715 and 646 admissions in the 30 months post-PCV10, respectively. Pneumonia hospitalizations declined by 37.8% (95% confidence interval [CI] 21.4–50.3%) and 28.8% (95% CI 17.7–38.7%) among children aged <1 year and 1–4 years, respectively, while meningitis hospitalizations declined by 72.1% (95% CI 63.2–79.0%) and 61.6% (95% CI 50.4–70.8%), respectively, in these age groups. In contrast, at the referral hospital, pneumonia hospitalizations remained stable and a smaller but significant decline in meningitis was observed among children aged 1–4 years (39.3%, 95% CI 16.2–57.5%).

**Conclusions:**

PCV10 introduction was associated with declines in meningitis and pneumonia hospitalizations in Zambia, especially in first-level care hospitals.

## Teaser

We evaluated the impact of the 2013 introduction of a 10-valent pneumococcal conjugate vaccine (PCV10), using hospital administrative data in Zambia. Substantial declines in pneumonia and meningitis hospitalizations among children aged <5 years were observed post-PCV10.

*Streptococcus pneumoniae* (pneumococcus) can cause severe infections, such as meningitis, pneumonia, and sepsis, in children [1]. In 2008, the World Health Organization (WHO) estimated that over half a million deaths due to pneumococcal disease occurred globally in children <5 years of age, with 89.4% of these deaths being related to pneumonia [2]. The highest burden of pneumococcal deaths are in sub-Saharan Africa and Southeast Asia [3, 4]. Human immunodeficiency virus (HIV) is an important risk factor for pneumococcal disease [5]. Low-income countries with high HIV prevalence can face a tremendous burden of invasive pneumococcal disease. A safe and effective pneumococcal conjugate vaccine (PCV) was first licensed in 2000 by the US Food and Drug Administration and has been widely used in high-income countries for almost 2 decades [6, 7]. In 2007, after 2 large clinical trials in Africa demonstrated the efficacy of PCV beyond North America and Europe, the WHO, following the recommendations from the Strategic Advisory Group of Experts on Immunization, adopted a policy that all countries should introduce PCV into their infant immunization programs [8–10]. With support from Gavi, the Vaccine Alliance, many low-income countries in Africa started introducing PCV, largely after 2010 [11].

Population-level PCV impact data on pneumonia and meningitis hospitalizations in African countries that have recently introduced PCV are limited. Population-based surveillance data from the Gambia, an early adopter of PCV in Africa, showed reductions of 24% and 63%, respectively, in the incidences of radiologically confirmed pneumonia with consolidation and of hypoxic pneumonia among children aged <5 years after PCV introduction [12]. However, the collection of robust, population-based surveillance data is resource-intensive and difficult to implement in many countries. Therefore, the use of administrative data to measure vaccine impact has become attractive. In Rwanda, where, similar to the Gambia, PCV7 was introduced in 2009 and replaced by PCV13 in 2011, hospital administrative data were used for the first time in Africa to measure PCV impact on pneumonia and meningitis hospitalizations, and demonstrated declines similar to those reported from population-based surveillance systems in the Gambia and Malawi [12–14]. In high-income countries, hospital administrative data have provided useful information on PCV impact and have been widely used [15–17].

Zambia, a country located in Southern Africa with an HIV prevalence of 12% among women attending antenatal care [18], introduced 10-valent PCV (PCV10) into the routine infant immunization program in July 2013, using a schedule of 3 doses at 6, 10, and 14 weeks of age, with no booster dose (3 + 0 schedule) and no catch-up campaign. Based on WHO and United Nations International Children’s Emergency Fund estimates, PCV10 coverage for 3 doses in Zambia increased from 77% in 2014 to 90% in 2016 [19]. We used hospital administrative data to evaluate the impact of PCV10 on pneumonia and meningitis hospitalizations in children <5 years of age in a country with a high HIV prevalence.

## METHODS

### Data Sources

Zambia is divided into 10 administrative provinces and has a total population of 13.1 million people, of whom 2.2 million are children <5 years of age [20]. Each province has at least 1 provincial general hospital, 5 to 15 district hospitals, and 1 to 182 urban health centers with 20–60 admission beds. Urban health centers serve a catchment population of between 30 000 and 50 000 people, while district hospitals serve catchment populations of between 80 000 and 200 000 people [21]. The flow of administrative data goes from the health facilities to the district health office, provincial health office, and, finally, to the Ministry of Health (MOH) Headquarters. We used District Health Information Software (DHIS2), an open-source health management information platform, to obtain meningitis and pneumonia hospitalization data from urban health centers, including 3 health centers in Lusaka that were upgraded to district hospitals (Chawama, Kanyama, and Chipata) in 2017 and that reported to MOH. No data from secondary- or tertiary-level care hospitals were available in DHIS2.

The University Teaching Hospital (UTH), a 2000-bed facility located in Lusaka, is the largest referral hospital in Zambia, providing tertiary-level care to patients coming from all over the country. UTH has built capacity in several specialty areas, including pulmonary medicine, intensive care, cardiac surgery, and orthopedics. UTH’s Department of Pediatrics and Child Health (now Lusaka Children’s Hospital) has a 400-bed capacity and over 10 000 admissions annually for children <5 years of age. Approximately 85% of UTH admissions are referred from other hospitals because of either clinical severity or the need for either specialized services, such as pulmonary medicine, or imaging studies, such as computed tomography. At UTH, after a patient’s discharge or death, their medical records are sent to the Health Management Information System Department, where discharge codes are assigned based on the clinical diagnosis at the time of discharge or death and are entered into an Excel database.

### Definitions

For the MOH data, all discharges and deaths reported under the DHIS2 standardized field of “respiratory infection pneumonia” or “meningitis” for children aged <1 year and 1 to 4 years were captured by month and year of discharge or death from 2010 through 2016.

For UTH, 10th revision of the International Statistical Classification of Diseases and Related Health Problems (ICD-10) codes were used to capture pneumonia and meningitis discharges or in-hospital deaths by month and year from 2010 through 2016 among children aged <1 year and 1 to 4 years. Although there are several ICD-10 codes available for all-cause pneumonia and all-cause meningitis, UTH used only ICD-10 codes J18.9 (pneumonia, unspecified organism) and G03.9 (meningitis, unspecified) for pneumonia and meningitis, respectively. Data for all admissions for children <5 years of age at UTH were obtained.

The pre-PCV10 period was defined as the period from January 2010 through June 2013, while the post-PCV10 period was defined as the period from July 2014 through December 2016. The period from July 2013 through June 2014 was excluded as a transition year with a low vaccine uptake.

### Validation of Discharge Codes

A review of medical records coded as pneumonia and meningitis from children <5 years of age was performed at UTH to determine the positive predictive value (PPV) of the ICD-10 codes in capturing our outcomes of interest. Trained sixth-year medical students abstracted relevant clinical, laboratory, and radiological information from a convenience sample of 90 medical records coded as J18.9 and 61 medical records coded as G03.9 from the pre- and post-PCV10 periods, using a standardized abstraction form. During the abstraction process, the lack of a particular finding in the medical record was treated as no evidence for that finding, rather than as missing information. An infectious diseases physician reviewed all the abstraction forms to determine the final diagnoses, based on a priori definitions for pneumonia and meningitis. The case definition for a final pneumonia diagnosis included either (1) respiratory findings (ie, cough, shortness of breath, or hypoxia) with a radiologic finding listed in the medical record as pneumonia, consolidation, pleural effusion, or a lung abscess; or (2) clinical documentation of pneumonia on the discharge summary. However, due to the absence of radiology reports or chest X-ray films during medical record reviews, only the latter definition was applied. Final meningitis diagnoses were based on the clinical documentation of meningitis on a discharge summary or meningeal signs in a child with a history of fever. We calculated the 95% confidence interval (CI) for the PPV using Wilson’s formula, as previously described [22].

### Data Analysis

We fit the monthly case counts during the pre-PCV10 period to negative binomial regression models. Separate models were developed for each outcome and age group (<1 and 1–4 years old). Hospitalization data based on MOH and UTH data were analyzed separately. The models included a continuous term to estimate the percent change in case counts by month and harmonic terms to capture the seasonal pattern of the case counts. The parameters estimated from the pre-vaccine time-series model and their variance provided the predictive distribution for post-PCV10 time-series model parameters. At each month, from July 2014 through December 2016, we calculated 1000 predicted case counts based on 1000 random draws from the predictive distribution of the model parameters. The median number of cases derived from those simulations represented the number of pneumonia or meningitis discharges or deaths expected in the absence of PCV10. The 95% confidence intervals around the point estimates represent the 2.5th and 97.5th percentiles of the simulations. The number of hospitalization or deaths averted was calculated as the difference between what was observed and what would have been expected in the absence of PCV10. The decline in hospitalizations or deaths was calculated as the ratio of the observed to the expected data in the post-PCV10 period. Because data on other disease conditions were only available for UTH, we performed an additional analysis for the UTH pneumonia models, using nonpneumonia, nondiarrhea (rotavirus vaccine was introduced in 2013), and nonbronchiolitis hospitalizations as control conditions. In addition to the seasonal harmonic terms and the continuous indicator, the adjusted UTH pneumonia models included monthly hospitalization counts for the control conditions and an interaction term between the control hospitalization counts and the continuous time indicator.

Chi-square test was used to compare case fatality ratios between pre- and post-PCV10 periods by age group.

This project was reviewed in accordance with Centers for Disease Control and Prevention and WHO human research protection procedures and was determined to be a nonresearch study. The protocol was also reviewed by the Ethical Review Committee from Zambia MOH and determined to be a public health evaluation.

## RESULTS

### Pneumonia Hospitalizations

**Figure 1.**
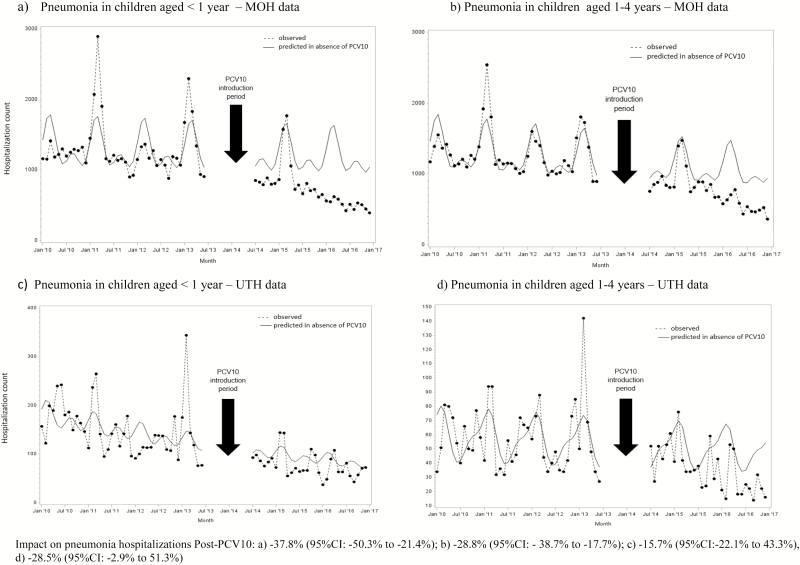
Modeled and observed pneumonia hospitalization counts by age and month/year of discharge using MOH and UTH data, Zambia, 2010–2016. Abbreviations: MOH, Ministry of Health; PCV10, 10-valent pneumococcal conjugate vaccine; UTH, University Teaching Hospital.

**Figure 2.**
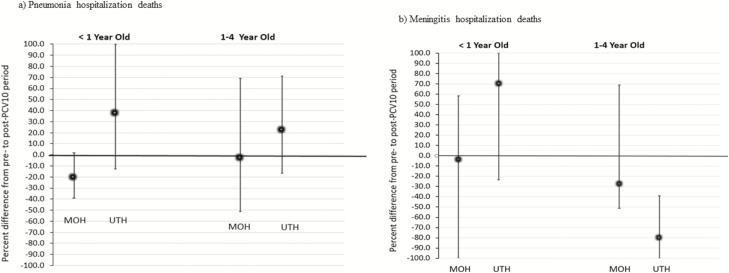
Estimated reductions in in-hospital pneumonia and meningitis deaths among hospitalized children aged <5 years post-PCV10 using MOH and UTH data, Zambia, 2010–2016. *A*, Pneumonia hospitalization deaths. *B*, Meningitis hospitalization deaths. Abbreviations: MOH, Ministry of Health; PCV10, 10-valent pneumococcal conjugate vaccine; UTH, University Teaching Hospital.

In the pre-PCV10 period (42 months), 54 706 and 54 178 pneumonia hospitalizations among children aged <1 year and 1–4 years, respectively, were reported to MOH; in the post-PCV10 period (30 months), 21 975 and 22 740 pneumonia hospitalizations among these same age groups were reported, respectively. Pneumonia hospitalizations peaked from January through April, which represents the rainy season in Zambia (Figure 1). Among infants, pneumonia hospitalizations declined by 37.8% (95% CI 21.4–50.3%), with an estimated 13 348 (95% CI 5989–22 223) hospitalizations averted after PCV10 introduction (Figure 1A). For children aged 1–4 years, a 28.8% (95% CI 17.7–38.7%) decline in pneumonia hospitalizations was observed, leading to 9204 (95% CI 4902–14 378) hospitalizations averted in the post-PCV10 period (Figure 1B). The in-hospital case-fatality ratio for pneumonia went from 6.5% in the pre-PCV10 period to 5.9% in the post-PCV10 period for children aged <1 year (*P* = .001) and from 3.5% pre-PCV10 to 3.1% post-PCV10 for children aged 1–4 years (*P* = .001). However, based on the time-series models, no significant changes in the number of in-hospital pneumonia deaths after PCV10 introduction were observed (Figure 2A).

For UTH, there were a total of 6306 and 2328 pneumonia hospitalizations among children aged <1 year in the pre- and post-PCV10 periods, respectively. Among children aged 1–4 years, 2412 and 1072 pneumonia hospitalizations pre- and post-PCV10 were reported, respectively. The in-hospital case-fatality ratios for pneumonia in infants were similar between the pre-PCV10 and post-PCV10 periods (15.3% vs. 16.3%, respectively; *P* = .2), while for children aged 1–4 years, the ratio increased from 8.8% pre-PCV10 to 15.2% post-PCV10 (*P* < .001). No significant changes in pneumonia hospitalizations were observed after the PCV10 introduction for either age group on unadjusted (Figure 1C and 1D) and adjusted time-series models (not shown). No declines in pneumonia deaths were observed based on the time-series models (Figure 2A).

### Meningitis Hospitalizations

**Figure 3.**
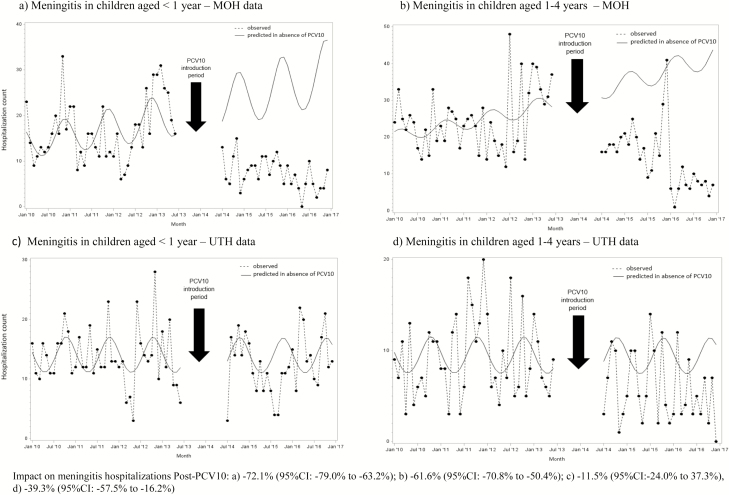
Modeled and observed meningitis hospitalization counts by age and month/year of discharge using MOH and UTH data, Zambia, 2010–2016. Impact on pneumonia hospitalizations post-PCV10: (*A*) −72.1% (95% CI −79.0 to −63.2%); (*B*) −61.6% (95% CI −70.8 to −50.4%); (*C*) −11.5% (95% CI −24.0 to 37.3%); and (*D*) −39.3% (95% CI −57.5 to −16.2%). Abbreviations: CI, confidence interval; MOH, Ministry of Health; PCV10, 10-valent pneumococcal conjugate vaccine; UTH, University Teaching Hospital.

Among children aged <1 year, 699 and 219 meningitis hospitalizations were reported to MOH pre- and post-PCV10 introduction, respectively. Among children aged 1–4 years, 1043 and 427 hospitalizations were reported pre- and post-PCV10 introduction, respectively. The in-hospital case-fatality ratios for meningitis among children aged <1 year were similar in the pre- and post-PCV10 periods (14.9% vs. 19.6%, respectively; *P* = .09), while for children aged 1–4 years the ratio decreased from 12.3% to 8.6%, respectively (*P* = .04). Meningitis hospitalizations declined by 72.1% (95% CI 63.2–79.0%) among children aged <1 year, with an estimated 567 (95% CI 377–825) hospitalizations averted in the post-PCV10 period. Among children aged 1–4 years, a decline of 61.6% (95% CI 50.4%–70.8%) was observed, with an estimated 686 (95% CI 433–1036) meningitis hospitalizations averted (Figure 3A and 3B). No significant declines in the number of meningitis deaths were observed in either age group based on MOH data (Figure 2B).

For UTH, there were a total of 685 and 426 meningitis hospitalizations among children aged <1 year in the pre- and post-PCV10 periods, respectively. Among children aged 1–4 years, 459 and 196 meningitis hospitalizations pre- and post-PCV10 introduction were reported, respectively. The in-hospital case-fatality ratio for meningitis in infants declined from 16.1% in the pre-PCV10 period to 11.7% in the post-PCV10 period (*P* = .04), while for children aged 1–4 years, it declined from 15.5% in the pre-PCV10 period to 10.2% in the post-PCV10 period, but the latter decline was not statistically significant (*P* = .07). Meningitis hospitalizations at UTH remained stable over time among children aged <1 year despite the PCV10 introduction, while among children aged 1–4 years it declined by 39.3% (95% CI 16.2–57.5%), leading to 122 (95% CI 34–235) meningitis hospitalizations averted in this age group (Figure 3C and 3D). The decline in meningitis cases in children 1–4 years of age was mainly observed after April 2016. Significant declines in in-hospital meningitis deaths were also observed for children aged 1–4 years at UTH (Figure 2B).

### Validation of Administrative Data

Of the 90 medical records reviewed with a discharge code of pneumonia, 32 (35.6%) were from the pre-PCV10 period and 58 (64.4%) were from the post-PCV10 period. The PPV of the pneumonia code (J18.9) was 90.6% (95% CI 73.8 –97.6%) in the pre-PCV10 period and 86.2% (95% CI 74.1–93.44%) in the post-PCV10 period.

For meningitis, 61 medical records were reviewed: 13 (21.3%) from the pre-PCV10 period and 48 (78.7%) from the post-PCV10 period. The PPV of the meningitis code (G03.9) was 100% (95% CI 71.7–100%) in the pre-PCV10 period and 89.6% (95% CI 76.5–96.1%) in the post-PCV10 period.

## DISCUSSION

We observed substantial declines in pneumonia and meningitis hospitalizations among children aged <5 years within 3 years after PCV10 introduction in Zambia. These declines were more pronounced among children hospitalized in first-level care hospitals, compared to those hospitalized at the largest tertiary care hospital in Zambia. Pneumonia hospitalizations at first-level care hospitals declined by 38% among infants and by 29% among children aged 1–4 years. These declines are similar to what have been reported, using either administrative data or active surveillance data, from other African countries, such as the Gambia and Rwanda, after PCV introduction, as well as in the United States after the introduction of PCV7 into a PCV-naive population [12–14, 23, 24]. Peaks in pneumonia hospitalizations were observed during the rainy season, which is consistent with what has been reported for invasive pneumococcal disease in Mozambique and is likely related to increased transmission due to household crowding during the rainy season [25]. The peaks in pneumonia are different from those for influenza or respiratory syncytial virus infection, which usually occur during the cool, dry season from March through August [26, 27].

Although an estimated 22 552 pneumonia hospitalizations were averted after the PCV10 introduction, no significant declines in the number of deaths among those hospitalized with pneumonia were observed. As expected, a lower case-fatality ratio was observed among children hospitalized with pneumonia at first-level care hospitals, compared to those hospitalized at UTH. Children hospitalized at UTH are likely to be more ill on admission and have more complex medical histories, compared to those hospitalized in first-level care hospitals. Case-fatality ratios differed by age, with a higher case-fatality ratio for pneumonia cases in infants, compared to children aged 1–4 years. The case-fatality ratios we observed for infants post-PCV10 introduction at both first-level care hospitals (5.9%) and UTH (16.3%) were similar to or higher than the 5.8% case-fatality ratio reported in Rwanda among children hospitalized with severe pneumonia [13]. In the post-PCV10 period, we observed a 58% increase in the case-fatality ratio for children aged 1–4 years who were hospitalized with pneumonia at UTH. Although the reason for this increase is unclear, it may indicate that, as first- and secondary-level care hospitals improve their capacity to admit and treat severe pneumonia, the patients referred to tertiary care hospitals such as UTH may be extremely ill. Declines in pneumococcal pneumonia deaths after PCV introduction have been documented [14, 28]. In our analysis, we measured deaths for all-cause pneumonia in hospitalized children, and it is unclear what proportion of these deaths are attributable to pneumococcal pneumonia. Therefore, the lack of decline we found for all-cause pneumonia deaths after the PCV10 introduction may be related to the low specificity of the outcome used in the analysis, in addition to the inclusion of death only among hospitalized children and the lack of power, given that death is a rarer outcome than pneumonia hospitalization.

Meningitis hospitalizations at the first-level care hospitals declined by 72% and 62% among infants and children aged 1–4 years, respectively, while a decline of 39% was observed for children aged 1–4 years who were hospitalized at UTH. Although the declines we observed in meningitis hospitalizations in the first-level care hospitals are higher than the 42% reduction reported in Rwanda using hospital administrative data, it falls within the range of the declines reported from countries in South America [13, 29]. It is possible that *S. pneumoniae* represents the largest burden of suspected bacterial meningitis, given that Zambia introduced a *Haemophilus influenzae* serotype B (Hib) vaccine back in 2004 and has maintained a coverage for the 3 doses of Hib of over 80% [11,19]. Studies conducted in African countries that have used both culture and molecular detection methods and that have introduced a Hib vaccine have found that up to 33% of suspected bacterial meningitis cases are due to *S. pneumoniae* [30–32]. However, many of these studies were conducted after a PCV introduction. Studies prior to a PCV introduction in Africa have relied heavily on culture methods, which likely underestimate the proportion of suspected bacterial meningitis attributable to *S. pneumoniae*. The large declines in meningitis hospitalizations we observed may also be related to interventions other than PCV in Zambia, such as increased coverage of programs for the prevention of mother-to-child HIV transmission, with widespread uptake of antiretroviral therapy by pregnant and breastfeeding women, which have resulted in substantial declines in the HIV prevalence among children after 2011 [33].

The case-fatality ratio we observed for meningitis was similar for infants across levels of care and was as high as 19.6% post-PCV10 introduction, suggesting a high severity of illness in patients with a diagnostic code of meningitis. In contrast to first-level care hospitals, the case-fatality ratios for meningitis hospitalizations at UTH were similar between infants and children aged 1–4 years (14.4% vs. 13.8%, respectively). Declines in the case-fatality ratio for meningitis from the pre- to post-PCV10 period were observed for infants at UTH and for children aged 1–4 years across levels of care. A time-series analysis showed significant declines in in-hospital meningitis deaths among children aged 1–4 years at UTH after the PCV10 introduction.

Our analyses have several limitations. First, medical record coding practices can change over time, which likely affects disease trends. Second, the absence of specific codes for pneumococcal disease led to the use of nonspecific outcomes, such as all-cause pneumonia and all-cause meningitis, which poses difficulties in the evaluation of trends over time. It is also possible that declines are more difficult to assess using referral hospital data, given that tertiary-care referral hospitals serve large geographic areas and referral patterns may change over time. Third, we were only able to obtain hospitalization data from healthcare facilities reporting to the MOH, which did not include any secondary-level care hospital, and from 1 tertiary care hospital. Therefore, the declines we observed may not be generalizable. Fourth, data on PCV immunization among hospitalized children were not available. Fifth, we have not assessed the sensitivity of the discharge codes for either pneumonia or meningitis, and it is possible that we may have missed hospitalizations for either of these diagnoses. Finally, interventions other than PCV after 2013 in Zambia may have resulted in declines in pneumonia and meningitis hospitalizations and deaths. Although the declines we observed in pneumonia hospitalizations were similar to those in other observational studies, the declines we observed in meningitis at first-level care hospitals were much higher than other reports from Africa. We were only able to validate the discharge codes at UTH; thus, the PPV of the medical records coded as meningitis at the first-level care hospitals is unknown and some misclassification may have occurred.

In summary, we demonstrated substantial declines in pneumonia and meningitis hospitalizations in children aged <5 years who were hospitalized at first-level care hospitals. In the 30 months following the PCV10 introduction in Zambia, an estimated reduction in 22 000 pneumonia and 1300 meningitis hospitalizations occurred. Our analysis shows that relying solely on administrative data from tertiary-care referral hospitals may not be as useful as analyses using data from multiple lower-level care hospitals. This is the first PCV impact evaluation in Zambia. It uses an approach that is subject to several limitations, but is relatively inexpensive for measuring the impact of a public health intervention. As countries in Africa fully implement the DHIS2 platform and more healthcare facilities in the countries start using this platform, these types of evaluation, using administrative data, may become more common. The results of our analysis may help inform vaccine policymakers in Zambia in considering the sustained use of PCV10 in the infant immunization program. Our approach can also be useful in the future to monitor whether the declines observed will be sustained.

## Notes

***Acknowledgments*.** The authors thank the University Teaching Hospitals, Children’s Hospital, Lusaka, Zambia, for providing a conducive hospital environment with a functional Health Management Information System e-database; the Ministry of Health, Directorate of Public Health & Research, HMIS unit for making available their national e-database; the World Health Organization Regional Office for Africa, Centers for Disease Control and Prevention, Gavi, the Vaccine Alliance, and other partners for generous support in providing the PCV10 vaccine and impact analysis to the Republic of Zambia; and Ministry of Health, Zambia, for the timely introduction of PCV10 in the Expanded Program on Immunizations program and their commitment in reducing childhood morbidity and mortality.

***Financial support***. This work was supported by Gavi, The Vaccine Alliance, through the CDC Foundation (grant number # 971-17).

***Supplement sponsorship.*** This supplement was supported with funds from Gavi, the Vaccine Alliance through The World Health Organization and the CDC Foundation, and The Medical Research Council Unit The Gambia at the London School of Hygiene and Tropical Medicine.

***Potential conflicts of interest.*** The authors: No reported conflicts of interest. All authors have submitted the ICMJE Form for Disclosure of Potential Conflicts of Interest. Conflicts that the editors consider relevant to the content of the manuscript have been disclosed.

## References

[1] American Academy of Pediatrics. Pneumococcal infections. In: Pickering LK, Baker CJ, Long SS, McMillan JA, eds. Red book: 2006 report of the Committee on Infectious Diseases. 27th ed Elk Grove Village, Illinois: American Academy of Pediatrics, 2006:525–537.

[2] World Health Organization. Estimated Hib and pneumococcal deaths for children under 5 years of age, 2008. Available at: http://www.who.int/immunization/monitoring_surveillance/burden/estimates/Pneumo_hib/en/. Accessed 19 November 2018.

[3] Walker CLF, Rudan I, Liu L, et al. Global burden of childhood pneumonia and diarrhoea. Lancet 2013; 381:1405–16. 23582727 10.1016/S0140-6736(13)60222-6 PMC7159282

[4] GBD 2016 Lower Respiratory Infections Collaborators. Estimates of the global, regional, and national morbidity, mortality, and aetiologies of lower respiratory infections in 195 countries, 1990–2016: a systematic analysis for the Global Burden of Disease Study 2016. Lancet Infect Dis 2018; 18:1191–1210. doi: 10.1016/S1473-3099(18)30310-4 30243584 PMC6202443

[5] van Aalst M, Lötsch F, Spijker R, et al. Incidence of invasive pneumococcal disease in immunocompromised patients: a systematic review and meta-analysis. Travel Med Infect Dis 2018; 24:89–100. 29860151 10.1016/j.tmaid.2018.05.016

[6] Van Beneden CA, Whitney CG, Levine OS, Schwartz B, Advisory Committee on Immunization Practices Working Group on Pneumococcal Conjugate Vaccine. Preventing pneumococcal disease among infants and young children: recommendations of the Advisory Committee on Immunization Practices (ACIP). MMWR Morb Mortal Wkly Rep 2000; 49:1–38.

[7] Choi YH, Jit M, Gay N, et al. 7-Valent pneumococcal conjugate vaccination in England and Wales: is it still beneficial despite high levels of serotype replacement? PLOS One 2011; 6:e26190. 22022559 10.1371/journal.pone.0026190 PMC3193519

[8] Klugman KP, Madhi SA, Huebner RE, Kohberger R, Mbelle N, Pierce N; Vaccine Trialists Group A trial of a 9-valent pneumococcal conjugate vaccine in children with and those without HIV infection. N Engl J Med 2003; 349:1341–8. 14523142 10.1056/NEJMoa035060

[9] Cutts FT, Zaman SM, Enwere G, et al; Gambian Pneumococcal Vaccine Trial Group Efficacy of nine-valent pneumococcal conjugate vaccine against pneumonia and invasive pneumococcal disease in the Gambia: randomised, double-blind, placebo-controlled trial. Lancet 2005; 365:1139–46. 15794968 10.1016/S0140-6736(05)71876-6

[10] World Health Organization. Pneumococcal conjugate vaccine for childhood immunization--WHO position paper. Wkly Epidemiol Rec 2007; 82:93–104. 17380597

[11] International Vaccine Access Center (IVAC), Johns Hopkins Bloomberg School of Public Health, View-hub report:global vaccine introduction and implementation. Available at: https://www.jhsph.edu/ivac/wp-content/uploads/2018/08/VIEW-hub_Report_Jun2018.pdf. Accessed 19 November 2018.

[12] Mackenzie GA, Hill PC, Sahito SM, et al. Impact of the introduction of pneumococcal conjugate vaccination on pneumonia in the Gambia: population-based surveillance and case-control studies. Lancet Infect Dis 2017; 17:965–73. 28601421 10.1016/S1473-3099(17)30321-3 PMC5589209

[13] Gatera M, Uwimana J, Manzi E, et al. Use of administrative records to assess pneumococcal conjugate vaccine impact on pediatric meningitis and pneumonia hospitalizations in Rwanda. Vaccine 2016; 34:5321–8. 27639280 10.1016/j.vaccine.2016.08.084

[14] McCollum ED, Nambiar B, Deula R, et al Impact of the 13-valent pneumococcal conjugate vaccine on clinical and hypoxemic childhood pneumonia over three years in central Malawi: an observational study. PLOS One 2017; 12:e0168209. 28052071 10.1371/journal.pone.0168209 PMC5215454

[15] van Deursen AMM, Schurink-Van’t Klooster TM, Man WH, et al. Impact of infant pneumococcal conjugate vaccination on community acquired pneumonia hospitalization in all ages in the Netherlands. Vaccine 2017; 35:7107–13. 29146381 10.1016/j.vaccine.2017.10.090

[16] Griffin MR, Zhu Y, Moore MR, Whitney CG, Grijalva CG U.S. hospitalizations for pneumonia after a decade of pneumococcal vaccination. N Engl J Med 2013; 369:155–63. 23841730 10.1056/NEJMoa1209165 PMC4877190

[17] Simonsen L, Taylor RJ, Schuck-Paim C, Lustig R, Haber M, Klugman KP Effect of 13-valent pneumococcal conjugate vaccine on admissions to hospital 2 years after its introduction in the USA: a time series analysis. Lancet Respir Med 2014; 2:387–94. 24815804 10.1016/S2213-2600(14)70032-3

[18] Central Statistical Office, Ministry of Health Zambia demographic and health survey 2013–2014. Available at: https://dhsprogram.com/pubs/pdf/FR304/FR304.pdf. Accessed 26 November 2018.

[19] World Health Organization. WHO and UNICEF Coverage Estimates by Country. Available at: http://apps.who.int/immunization_monitoring/globalsummary/timeseries/tswucoveragedtp3.html. Accessed 19 November 2018.

[20] Central Statistical Office, Republic of Zambia 2010 Zambia census of population and housing: population summary report. Available at: http://www.zamstats.gov.zm/. Accessed 19 November 2018.

[21] Institute for Health Metrics and Evaluation, University of Washington and University of Zambia. Health service provision in Zambia. Available at: http://www.healthdata.org/sites/default/files/files/policy_report/2015/ABCE_Zambia_finalreport_Jan2015.pdf. Accessed 19 November 2018.

[22] Niesner K, Murff HJ, Griffin MR, et al. Validation of VA administrative data algorithms for identifying cardiovascular disease hospitalization. Epidemiology 2013; 24:334–5. 23377095 10.1097/EDE.0b013e3182821e75 PMC4667547

[23] Izu A, Solomon F, Nzenze SA, et al Pneumococcal conjugate vaccines and hospitalization of children for pneumonia: a time-series analysis, South Africa, 2006–2014. Bull World Health Organ 2017; 95:618–28. 28867842 10.2471/BLT.16.187849 PMC5578378

[24] Grijalva CG, Nuorti JP, Arbogast PG, Martin SW, Edwards KM, Griffin MR Decline in pneumonia admissions after routine childhood immunisation with pneumococcal conjugate vaccine in the USA: a time-series analysis. Lancet 2007; 369:1179–86. 17416262 10.1016/S0140-6736(07)60564-9

[25] Roca A, Sigaúque B, Quintó L, et al. Invasive pneumococcal disease in children <5 years of age in rural Mozambique. Trop Med Int Health 2006; 11:1422–31. 16930265 10.1111/j.1365-3156.2006.01697.x

[26] Saijo M, Terunuma H, Mizuta K, et al. Respiratory syncytial virus infection in children with acute respiratory infections in Zambia. Epidemiol Infect 1998; 121:397–400. 9825792 10.1017/s0950268898001228 PMC2809538

[27] Theo A, Liwewe M, Ndumba I, et al Influenza surveillance in Zambia, 2008–2009. J Infect Dis 2012; 206(Suppl 1):S173–S177. 23169966 10.1093/infdis/jis599

[28] Wahl B, O’Brien KL, Greenbaum A, et al. Burden of *Streptococcus pneumoniae* and *Haemophilus influenzae* type B disease in children in the era of conjugate vaccines: global, regional, and national estimates for 2000-15. Lancet Glob Health 2018; 6:e744–57. 29903376 10.1016/S2214-109X(18)30247-X PMC6005122

[29] de Oliveira LH, Camacho LA, Coutinho ES, et al. Impact and effectiveness of 10 and 13-valent pneumococcal conjugate vaccines on hospitalization and mortality in children aged less than 5 years in Latin American countries: a systematic review. PLOS One 2016; 11:e0166736. 27941979 10.1371/journal.pone.0166736 PMC5152835

[30] Gituro CN, Nyerere A, Ngayo MO, Maina E, Githuku J, Boru W Etiology of bacterial meningitis: a cross-sectional study among patients admitted in a semi-urban hospital in Nairobi, Kenya. Pan Afr Med J 2017; 28(Suppl 1):10. 10.11604/pamj.supp.2017.28.1.9383 PMC6113691 30167035

[31] Oordt-Speets AM, Bolijn R, van Hoorn RC, Bhavsar A, Kyaw MH Global etiology of bacterial meningitis: a systematic review and meta-analysis. PLOS One 2018; 13:e0198772. 29889859 10.1371/journal.pone.0198772 PMC5995389

[32] Nhantumbo AA, Cantarelli VV, Caireão J, et al. Frequency of pathogenic paediatric bacterial meningitis in Mozambique: the critical role of multiplex real-time polymerase chain reaction to estimate the burden of disease. PLOS One 2015; 10:e0138249. 26393933 10.1371/journal.pone.0138249 PMC4578858

[33] Price JT, Chi BH, Phiri WM, et al. Associations between health systems capacity and mother-to-child HIV prevention program outcomes in Zambia. PLOS One 2018; 13:e0202889. 30192777 10.1371/journal.pone.0202889 PMC6128540

